# P-2102. Performance of the VITEK®2 Advanced Expert System (AES) as a Rapid Aid for Reporting Antimicrobial Susceptibility Testing in *Pseudomonas aeruginosa* and *Acinetobacter* spp

**DOI:** 10.1093/ofid/ofae631.2258

**Published:** 2025-01-29

**Authors:** Meredith Hackel, Mary Bailey-Person

**Affiliations:** IHMA, Schaumburg, Illinois; IHMA, Schaumburg, Illinois

## Abstract

**Background:**

The VITEK^®^2 Advanced Expert System (AES) is an automated antibiotic susceptibility testing (AST) system which provides rapid AST data by interpreting and checking each MIC against a database of phenotypes and MIC distributions and infers a resistance mechanism. AES provides a level of confidence for auto-releasing reports of green (consistent), yellow (consistent with correction), and red (inconsistent, MIC pattern not matching any phenotype). This study evaluated VITEK^®^2 AES reports compared to broth microdilution (BMD) on a set of *Pseudomonas aeruginosa* (PA) and *Acinetobacter* spp. (ABC).

**Methods:**

A total 148 PA and 150 ABC were tested. Susceptibility was tested by VITEK^®^2 (N802 and XN15 AST cards) and CLSI reference BMD against 13 antimicrobials for PA and 17 antimicrobials for ABC applying 2021 CLSI breakpoints. Molecular characterization of isolates was based on WGS or PCR of β-lactamase genes. Permeability and gene expression were not evaluated.

**Results:**

Among 143 ABC, AES reports were green for 131 (91.6%), and yellow for 12 (8.4%) isolates. Green reports agreed with BMD for 131 isolates (100%) and could have been automatically reported without further review. Among yellow reports, AES corrections were acceptable for 11 isolates (91.7%). Among 40 carbapenem-producing ABC, AES reports were green for 39 isolates (97.5%) and yellow for one isolate (2.5%). Green reports agreed with BMD for 37 isolates (94.9%) and could have been automatically reported. Of 148 PA, AES reports were green for 125 (84.5%), yellow for 18 (12.2%), and red for 5 (3.4%) isolates. Of 112 PA with molecular data, 64 (57.1%), 38 (33.9%), 6 (5.4%), 3 (2.7%), and one (0.9%) contained genes for intrinsic β-lactamases only (WT), MBL, GES carbapenemase, KPC, and ESBL genes only, respectively. Overall, the AES accurately reported phenotypes for 97/112 PA (86.6%). Among the MBL-positive PA, 37/38 (97.4%) were reported as green and all showed AES phenotype agreement with the genotype. Among the WT PA, 51/64 (79.7%) were reported as green and 82.4% showed AES phenotype agreement with the genotype.

**Conclusion:**

The use of VITEK^®^2 AES for the confident release of AST results without additional review could have a positive impact on time savings in the laboratory and aid in antimicrobial stewardship.

**Disclosures:**

All Authors: No reported disclosures

